# Phasic pupillary responses modulate object-based attentional prioritization

**DOI:** 10.3758/s13414-020-02232-7

**Published:** 2021-01-27

**Authors:** Sean R. O’Bryan, Miranda Scolari

**Affiliations:** Department of Psychological Sciences, Texas Tech University, MS 2051, Lubbock, TX 79409 USA

**Keywords:** Object-based attention, Space-based attention, Attentional prioritization, Psychophysics, Pupillometry

## Abstract

Visual attention studies have demonstrated that the shape of space-based selection can be governed by salient object contours: when a portion of an enclosed space is cued, the selected region extends to the full enclosure. Although this form of object-based attention (OBA) is well established, one continuing investigation focuses on whether this selection is obligatory or under voluntary control. We attempt to dissociate between these alternatives by interrogating the locus coeruleus-norepinephrine (LC-NE) system – known to fluctuate with top-down attention – during a classic two-rectangle paradigm in a sample of healthy human participants (N = 36). An endogenous spatial pre-cue directed voluntary space-based attention (SBA) to one end of a rectangular frame. We manipulated the reliability of the cue, such that targets appearing at an uncued location within the frame occurred at low or moderate frequencies. Phasic pupillary responses time-locked to the cue display served to noninvasively measure LC-NE activity, reflecting top-down processing of the spatial cue. If OBA is controlled analogously to SBA, then object selection should emerge only when it is behaviorally expedient and when LC-NE activity reflects a high degree of top-down attention to the cue display. Our results bore this out. Thus, we conclude that OBA was voluntarily controlled, and furthermore show that phasic norepinephrine may modulate attentional strategy.

**Supplementary Information:**

The online version contains supplementary material available at 10.3758/s13414-020-02232-7.

## Introduction

Egly et al. (1994) introduced to the visual attention literature the now ubiquitous two-rectangle paradigm, which commonly elicits a selection of space governed by object contours. Since then, several object-based attention (OBA) studies have reported a reaction time (RT) benefit when a spatially invalid cue directs attention within – rather than outside of – the same object as an upcoming target (Atchley & Kramer, 2001; Behrmann, Zemel, & Mozer, 1998; Lamy & Egeth, 2002; Marrara & Moore, 2003; Moore et al., 1998; Watson & Kramer, 1999). Early research results using the two-rectangle paradigm were generally interpreted in support of an automatic spread of spatial attention, whereby all visual information within an object contour is selected for preferential processing even when only a small section of bounded space is behaviorally relevant (Abrams & Law, 2000; Egly et al., 1994).

**Fig. 1:**
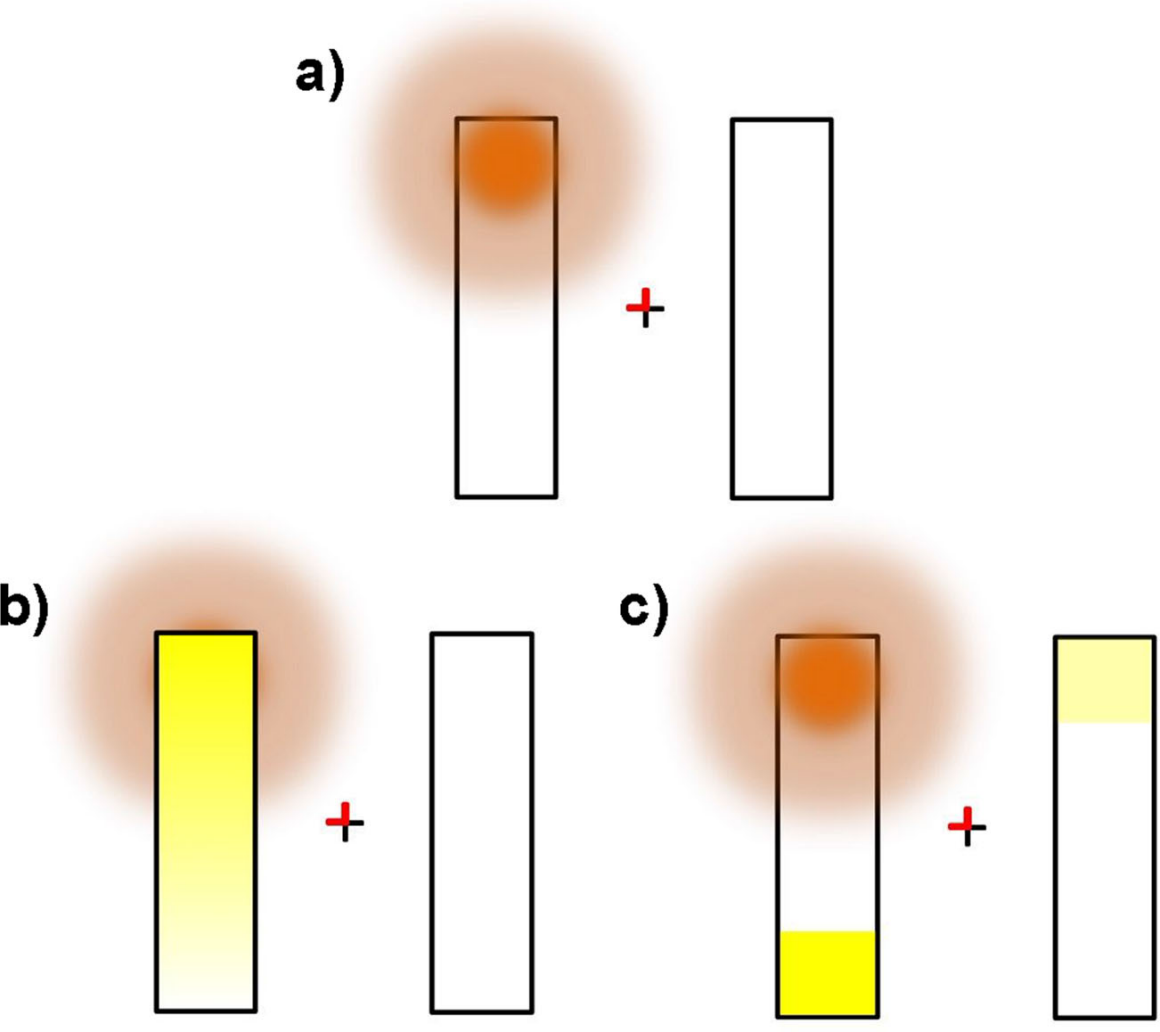
Theoretical accounts of space- and object-based attention following an endogenous pre-cue. More saturated colors indicate greater sensory enhancement, with orange corresponding to space-based effects, and yellow corresponding to object-based effects. **(a)** Spatial gradient only. Space-based attention is directed to the top end of the left rectangular frame via the endogenous cue (red line segments overlaid on the fixation cross). **(b)** Attentional spreading. **(c)** Attentional prioritization. Figure adapted from Fig. 2 of Shomstein (2012, p.164) with permission from John Wiley and Sons

Notably, though, in the traditional two-rectangle paradigm, targets occasionally appear at uncued spatial locations such that deploying attention to all probable target locations offers some performance benefits. As a result, at least two accounts have emerged to describe the distribution of space-based attention (SBA) across an object: (1) Consistent with early interpretations, SBA automatically spreads within an attended object’s contours (attentional spreading hypothesis; Chen & Cave, 2006, 2008), or (2) attention is strategically directed to probable target locations within a relevant object and without selection of intervening, nontarget locations (attentional prioritization hypothesis; Shomstein, 2012; Shomstein & Behrmann, 2008; Shomstein & Yantis, 2004, 2006). According to this alternative account, uncued target locations within a cued object are prioritized over other uncued locations via gestalt grouping mechanisms, thus resulting in a same-object benefit. Although both models can account for the same traditional pattern of results in the two-rectangle paradigm, they importantly make distinct predictions regarding how, why, and under what circumstances object contours shape spatial selection (see Fig. 1).

Shomstein and Yantis (2002) offered early support for attentional prioritization by flanking the target with a pair of either compatible or incompatible distractors, where all three elements were either positioned within common (same-object) or unique (different-object) rectangles. Although an automatic attentional spreading account would predict that the presence of an incompatible same-object distractor should result in a larger behavioral cost compared to its different-object counterpart, RT was not differentially impeded across conditions. Thus, OBA effects may not emerge when within-object locations are behaviorally irrelevant (but see Chen & Cave, 2006, 2008).

The predictions of the attentional spreading and prioritization accounts similarly diverge across manipulations of cue reliability. If participants voluntarily deploy attention to probable target locations, as the prioritization account argues, then RT patterns should scale with the probability that a target will appear in each respective location. Empirical evidence for this kind of attentional “probability matching” comes via studies that manipulated the likelihood of spatially invalid targets appearing in one of two fixed spatial locations: participants were faster to discriminate targets appearing in a location that included 87% of all invalid targets versus another location where only 13% of invalid targets appeared. Critically, the effects of this probability manipulation were found to be more robust than traditional object-based effects within the context of a two-rectangle paradigm (Shomstein & Behrmann, 2008; Shomstein & Yantis, 2004). OBA effects may only emerge, Shomstein and colleagues argue, when probabilistic imbalances across target locations and sufficiently salient object representations render such selection behaviorally expedient. For example, although Shomstein and Behrmann (2008) did not find OBA effects as consistently as probabilistic ones, the former emerged when the authors made the two rectangles saliently distinct by presenting them in different colors.

Apart from physical display changes, whether object contours are used to guide spatial selection may depend on the distribution of attention. In fact, such changes in how attention is distributed across a display do appear to impact whether and how object boundaries guide spatial selection (Al-Janabi & Greenberg, 2016). Goldsmith and Yeari (2003) demonstrated in a series of experiments that traditional OBA effects can be observed when task conditions presumably elicit spatially diffuse attention and eliminated when conditions conversely elicit a focused or narrow attentional distribution. Thus, tradeoffs between narrow and broad attentional distributions can modulate OBA effects. Although not explicitly tested, these findings furthermore suggest that the spatial extent of attention across a display may be flexible.

The flexibility alluded to above – that object boundaries may be filtered out to mitigate their impact on the shape of spatial selection (Shomstein & Yantis, 2004) or utilized to guide search (Shomstein, 2012), depending on which approach is most behaviorally expedient – likely implicates voluntarily controlled, top-down attention. Consistent with the notion that OBA is partially (if not wholly) under voluntary control, regions within the frontoparietal attentional control network are activated when full objects are preferentially selected at the exclusion of others (Hou & Liu, 2012; Serences et al., 2004; Shomstein & Behrmann, 2006; Wojciulik & Kanwisher, 1999). For example, Hou and Liu (2012) found differential patterns of activation in intraparietal sulcus (IPS) and the frontal eye field (FEF) when participants attended to one of two superimposed triangles to detect changes in luminance. Using an endogenous spatial cue in conjunction with a two-rectangle paradigm, Shomstein and Behrmann (2006) found that left posterior parietal cortex (PPC) showed greater activation following a cue to shift attention within an object than a between-object shift cue. Together, these studies suggest that OBA is directed by a similar, if not common, top-down control mechanism to SBA (Scolari et al., 2014; Scolari et al., 2015).

Although activation of the attentional control network is arguably a necessary finding for a top-down account of object-based spatial selection, the studies cited above do not rule out an attentional spreading account. In most cases, task demands required whole object selection, precluding a strong test of an automatic spread of attention (Hou & Liu, 2012; Serences et al., 2004; Wojciulik & Kanwisher, 1999). At the same time, the elevated BOLD response reported by Shomstein and Behrmann (2006) could either signal that the within-object location was designated as having high search priority (attentional prioritization account) or that the within-object location was automatically included within the attended field by way of sensory stimulation (attentional spreading account). Furthermore, the study produced no evidence of behavioral attention effects, precluding the authors from relating behavioral effects to activation strength. Thus, it remains unclear whether traditional OBA effects are invariably the result of a bottom-up process whereby attention is automatically distributed across whole objects, or whether these effects emerge as a result of voluntary attentional deployment.

Indices of activity in the locus coreuleus-norepinephrine (LC-NE) system may yield further insight into whether object-based spatial selection is under voluntary control. Norepinephrine modulates the strength of neural representations, serving as both a temporal and a spatial attentional filter (Aston-Jones & Cohen, 2005; Eldar et al., 2013; Eldar et al., 2016; Gilzenrat et al., 2010; Mather et al., 2016; Thiele & Bellgrove, 2018). Salient, high-priority information is afforded a representational gain via cortical NE release while non-salient information associated with weak signals are further inhibited, resulting in greater neural selectivity. In line with this model, NE has been shown to improve the precision of sensory responses in rat visual cortex, particularly for sensory populations representing subthreshold or perithreshold stimuli (Hurley et al., 2004; Waterhouse et al., 1998). Similarly, pharmacologically elevating NE levels in healthy human participants improved behavioral performance on thresholded detection and discrimination tasks (Gelbard-Sagiv et al., 2018).

Variability in the firing modes of LC-NE neurons are thought to reflect fluctuations in attentional state, such that phasic bursts of activity time-locked to stimuli relate to focused attention and optimal task performance (Aston-Jones & Cohen, 2005; Aston-Jones et al., 1994; Mittner et al., 2016; Rajkowski et al., 1994; Sara & Bouret, 2012). With relevance to the current study, recent evidence suggests that tradeoffs between NE-mediated control states are likely to affect performance on spatial cueing tasks. Importantly, manipulating the specificity of spatial cues affects the temporal onset of phasic LC-NE responses (Geva et al., 2013), and the amplitude of such responses appears to correlate with space-based validity effects in RT during a Posner cueing task (Gabay, Pertzov, & Henik, 2011). Though untested to date, a similar relationship may exist with object-based selection.

### Present study

Here, we address two related questions about the mechanisms that underlie OBA. First, we conducted a between-subjects manipulation of the reliability of a central, endogenous pre-cue in an otherwise identical two-rectangle display (Egly et al., 1994; Shomstein & Yantis, 2004) in an attempt to find support for either the attentional prioritization or attentional spreading accounts. This approach enables us to hold stimulus display parameters constant while stringently testing how target location uncertainty might modulate selection in the presence of object contours. If the prioritization account holds, we expect RTs to be faster on invalid trials for both same- and different-object locations under conditions of high uncertainty.

Whether probability matching is accompanied with OBA effects – operationally defined as faster RTs to same- versus different-object invalid targets – may depend on the degree to which attentional control is activated. To elucidate how and whether top-down control modulates object-based attention effects, we measured pupil diameter (PD) following cue presentation as an indirect and noninvasive index of phasic LC-NE activity. Converging evidence suggests that moment-to-moment fluctuations in PD and firing rates in the LC are tightly coupled (Aston-Jones & Cohen, 2005; Aston-Jones et al., 1994; Costa & Rudebeck, 2016; Joshi et al., 2016; Mittner et al., 2016; Rajkowski et al., 1994; Reimer et al., 2016), and the magnitude of task-evoked pupil dilation is positively correlated with attentional control demands (Eldar et al., 2013; Eldar et al., 2016; Gilzenrat et al., 2010; Hopstaken et al., 2015; for review, see Van Der Wel & Van Steenbergen, 2018). Accordingly, PD measures have been increasingly relied upon as a proxy to infer attentional selectivity driven by underlying LC-NE activity (Einhäuser et al., 2008; Eldar et al., 2013; Eldar et al., 2016; Gabay et al., 2011; Gilzenrat et al., 2010; Mittner et al., 2016). Because we focused specifically on the phasic pupillary response during the cueing period – in which all stimulus display properties were held constant across cueing conditions and cue reliability groups – only top-down control mechanisms should contribute to any representational gain differences between possible target locations (Eldar et al., 2016). We reasoned that if OBA deployment is contingent on voluntary attentional control, object-based effects should be enhanced in the event of relatively large pupillary responses to the cue, and, conversely, diminished in cases of relatively small pupillary responses when spatial cue validity is low and thus target location uncertainty is high. Alternatively, if OBA is driven solely by properties of the stimulus display, we would expect any relationship between this physiological response and the presence or magnitude of OBA effects to remain constant across cue reliability manipulations.

## Methods

### Participants

A power analysis based on the average magnitude of within-subject OBA effects across several related behavioral studies revealed a large mean effect size (estimated *d* = 0.81; Chen & Cave, 2008; Goldsmith & Yeari, 2003; Shomstein & Behrmann, 2008; Shomstein & Yantis, 2004), corresponding to a projected sample size of 12 participants per group for 80% power with a 5% false-positive rate for within-group OBA effects. For between-group effects, we estimated a large effect size of *d =* 1.04, uncorrected, by averaging across within-subject effect sizes for both OBA effects (Chen & Cave, 2008; Goldsmith & Yeari, 2003; Shomstein & Behrmann, 2008; Shomstein & Yantis, 2004) and target probability manipulations (Shomstein & Behrmann, 2008; Shomstein & Yantis, 2004). Under the assumption of a moderate within-subject correlation across previous studies (rho = 0.6), the effect size estimate was adjusted to *d* = 0.89, corresponding to a projected sample size of 20 per group to detect between-group effects with 80% power and a 5% false-positive rate.

Forty-two healthy volunteers (31 female, 11 male; age range: 19–47 years) with normal or corrected-to-normal vision were recruited to participate in two or three separate 2-h study sessions for $10/h. Participants were randomly assigned to high (*n* = 20) or low (*n* = 22) cue validity conditions (see *Materials and stimuli* below). Five participants (three from the high-validity group and two from the low-validity group) only completed one experimental session, and thus were excluded from the analysis due to insufficient data. One additional participant from the low spatial-validity group was excluded due to poor eye tracking, resulting in a final analyzed sample size of 36 (high validity: *n* = 17; low validity: *n* = 19). We further verified that the sample size used in the current study after exclusions is consistent with those reported in other object-based attention studies and pupillometry studies that we cite (Alnæs et al., 2014; Chen & Cave, 2006, 2008; Gabay et al., 2011; Goldsmith & Yeari, 2003; Shomstein & Behrmann, 2008; Shomstein & Yantis, 2002, 2004; range: 12–20 participants). Two participants who completed three sessions were unintentionally run in the incorrect condition for one session; only the remaining two sessions were thus included in the analyses. All participants provided written informed consent in accordance with the Declaration of Helsinki and protocols were approved by the Texas Tech University Institutional Review Board.

### Materials and stimuli

Visual stimuli were created using MATLAB (v.9.1) and presented via Psychophysics Toolbox (v.3.3; Kleiner et al., 2007) on a desktop PC running Windows 10. Stimuli were displayed on a 1,920 × 1,080 pixel resolution BenQ XL2430T monitor measuring 58 cm wide and set to a 100-Hz refresh rate. The luminance output of the computer monitor was measured with a Minolta LS-110 photometer and linearized in a custom MATLAB script.

Each stimulus display was presented on a middle gray background screen (luminance of 105.23 cd/m^2^) and included a set of large black (0 cd/m^2^) rectangular frames measuring 3° × 6° of visual angle. The rectangular frames were centered at a Euclidean distance of 4.5° away from a central black fixation cross (0.3° × 0.3°), either oriented vertically to the left and right, or oriented horizontally above and below fixation. The rectangular frames served as object contours, within which a single target appeared. The target, akin to a Landolt C, was the alphanumeric character C presented in dark gray and in font style Bauhaus 93 at a font size of 48 (0.56°). It was rotated either 0° or 180° such that the small gap (0.06°) was facing to either the left or the right. The luminance of the target was initialized to 92.61 cd/m^2^, and subsequently titrated according to each participant’s performance (see *Titration procedure* below). The target C was equally likely to be positioned at any one end of either rectangular frame, 1.5° from the closest vertical and horizontal edges of the frame, and at a distance of 4.74° from fixation. The alphanumeric character “O,” presented in the same font style, size, and color as the C, served as a backward mask to the target. The luminance of the mask was yoked to that of the target.

### Procedure

**Fig. 2:**
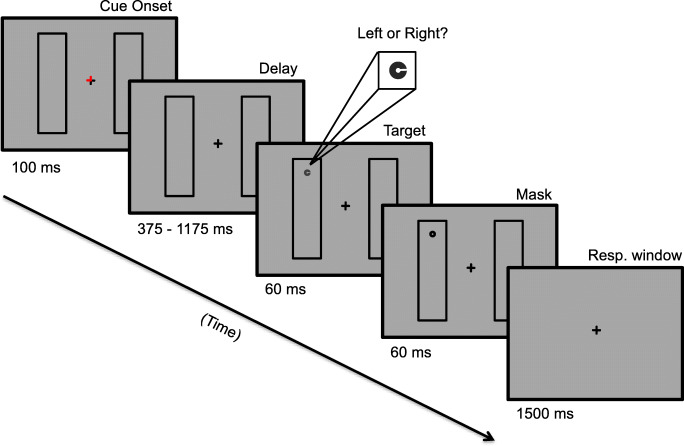
Stimuli and example of a valid trial from the modified two-rectangle task. The red line segments overlaid on fixation served as an endogenous location cue; in this example, directing attention to the top end of the left rectangular frame. After a variable delay, participants made a two-alternative forced choice decision about the rotation of the low-contrast target C

Participants were seated in a dark room at a distance of 92 cm from the computer monitor and instructed to maintain fixation on the black central cross. The study employed a modified version of the two-rectangle paradigm (Egly et al., 1994) using an endogenous spatial cue and a 2AFC target discrimination task (see Fig. 2). Trials began with a 100-ms red spatial cue overlaid on one of the four corners of the fixation cross to indicate the probable location of an upcoming target. The position of the cue was randomized across trials. At the time of cue onset, the two rectangular frames simultaneously appeared, oriented either horizontally or vertically as determined on a trial-by-trial basis. Following the onset of the cue and rectangles was a delay period, its duration randomly drawn from a distribution ranging from 300 to 1,175 ms in 25-ms steps. During the delay, both the fixation cross and rectangles remained on-screen. Participants did not know a priori precisely when the target would appear, and therefore were expected to anticipate its onset during the full length of the delay period.

The low-contrast target C then appeared for 60 ms at one of three possible locations relative to the cue: the valid (cued) location, the invalid (uncued) same-object location (contained within the boundaries of the cued rectangle), or the invalid different-object location (contained within the uncued rectangle but equidistant from the cued location relative to the same-object invalid location). Following a 10-ms interstimulus interval (ISI), a single O mask appeared in the target location for 60 ms.

The rectangles disappeared with mask offset and participants were given 1.5 s to report the orientation of the target C by pressing the left or right arrow key. Participants were instructed to respond as quickly and as accurately as possible, and both responses and RTs were recorded. Participants were given an on-screen readout of their mean accuracy during a brief break at the conclusion of each block. The task lasted for 16–24 total blocks (180 trials each), divided across two to three separate eight-block study sessions. Because the task was perceptually demanding, participants were offered brief breaks halfway through and between each block.

The purpose of this experiment was to investigate whether cue validity modulates object-mediated spatial selection. As such, participants were randomly assigned to a high or low spatial-validity condition. In the high spatial-validity condition, central pre-cues were valid on 80% of trials, with 10% of targets appearing in the invalid same-object location and 10% appearing in the different-object location. In the low spatial-validity condition, 50% of cues were valid, with the remaining 50% of targets equally distributed across the two possible invalid locations. Participants were explicitly informed about the proportion of valid- and invalid-cue trials associated with their assigned spatial validity condition.

### Titration procedure

Given that the task included a brief and perceptually challenging target, we employed a titration procedure to compensate for individual differences in perceptual ability. The goal of this procedure is to ensure that task difficulty did not vary across participants, both within and between spatial-validity conditions. Target contrast was initialized at 33% screen brightness relative to a middle gray background, with a contrast staircasing procedure implemented to maintain an accuracy range of 55% and 70% for each study session. This somewhat low performance criterion range was selected to ensure that the target would not become salient enough to pop-out against the gray background – hence, participants should be motivated to utilize the endogenous cue even during a long delay. Behavioral performance was evaluated every 15 trials; if mean accuracy over the last 15 trials fell below the criterion range, target contrast was increased by five linearized display units and was decreased by the same value if accuracy during this period exceeded the upper bound. Importantly, all trial types underwent a common titration procedure for each participant. Thus, although the overall participant mean was constrained to fall within the criterion range, there was no such restriction on how much performance on each trial type could deviate from the mean.

### Eye-tracking and pupillometry

Throughout the experiment, fixation was monitored using an EyeLink 1000 Plus infrared eye-tracker (SR Research, Ontario, Canada). The eye-tracker camera was positioned on a desk in front of the stimulus-presentation monitor at a distance of 55 cm from the participant’s forehead. Prior to the main experiment, the eye-tracker was calibrated for each participant using a 13-point grid and validated with an acceptability threshold of < 1° average cartesian prediction error. Eye position, velocity, eye blinks, and pupil diameter were sampled from the right eye at a rate of 500 Hz. Eye position relative to fixation was actively monitored by an experimenter in real time, and participants were instructed to re-direct their gaze to fixation in the event of a saccade or deviation from fixation.

Under carefully controlled conditions, pupil size has been demonstrated to be an appropriate indirect measure of phasic locus coeruleus norepinephrine (LC-NE) activity (Alnæs et al., 2014; Aston-Jones & Cohen, 2005; Jepma & Nieuwenhuis, 2011; Joshi et al., 2016; Reimer et al., 2016). We therefore used phasic pupil diameter to infer differences in pre-cue display processing, with relatively larger time-locked pupillary responses ostensibly associated with accurate processing of the cue and/or greater selective attention allocated to cued locations. Importantly, given the exceedingly brief target duration (60 ms; too short for an attentional shift during presentation), and variable delay, participants were expected to deploy and maintain attention during the cue period in anticipation of the target. Thus, response time to the target for each cueing condition should depend on the pattern of attentional deployment just prior to onset. We therefore investigated whether relative changes in pupil size between baseline (cue onset) and target onset predict the deployment of space- and object-based selection, either independently or interactively with the high and low spatial-validity conditions.

### Behavioral analyses

Before examining how phasic pupil diameter may modulate attention effects in the two-rectangle paradigm, we analyzed the behavioral data to (1) ensure that participants were using the endogenous spatial cue to guide their attention to the display, and (2) determine whether manipulating the reliability of the spatial cue affected how attention was distributed across valid, invalid same-object, and invalid different-object target locations. To calculate space- and object-based attention effects at the group and participant levels, we used performance on same-object invalid trials as the comparison condition. SBA was defined as significantly faster RTs to the valid versus invalid same-object location, whereas OBA was defined as significantly faster RTs to the invalid same-object location versus the invalid different-object location. This approach permits both space- and object-based attention effects to be observed independently (without influence of the other) and/or simultaneously.

#### Behavioral preprocessing

All behavioral analyses reported in the main text relate to RT on correct trials only. To ensure that our RT results were not influenced by a speed-accuracy tradeoff, we computed Pearson correlations between trial-by-trial RT and correct/incorrect responses for each subject, and tested the resulting r-values against a null hypothesis of zero correlation using a one-sample t-test.

Diagnostic plots revealed that correct RTs were characteristically positively skewed; accordingly, a square root transformation was performed to approximate a normal distribution across RT observations. Matching the preprocessing performed on the pupil data (see *Pupillometry preprocessing* below), RTs were then normalized within session for each participant via z-scoring to account for variability in mean RT across sessions and between participants (Faust et al., 1999; Hedge et al., 2018).

#### Statistical analyses

A linear mixed model with random intercepts for subjects and predictors (spatial validity condition, target location, and their interaction) modeled as fixed effects was first computed to test for behavioral effects of the manipulation using the “nlme” package in R (http://www.R-project.org/; R Core Team, 2014). Previous research using within-subject manipulations of target location probabilities suggests that RTs to invalid targets should become faster as the likelihood of targets appearing in a spatially invalid location increases (Shomstein, 2012; Shomstein & Behrmann, 2008). Accordingly, we predicted that participants in the low spatial-validity group would respond more quickly to invalid targets, consistent with flexible attentional prioritization. To test this prediction, we performed a two-sample t-test (unequal variances) to compare mean RTs on all invalid trials between the two spatial-validity groups. Additionally, we tested the prediction that object-based effects may be enhanced under conditions of increasing uncertainty. Thus, the results of the behavioral mixed model were decomposed into specific SBA and OBA effects using paired t-tests within each spatial-validity group, contrasting mean RTs between valid trials and same-object invalid trials (SBA effect), and between different-object invalid trials and same-object invalid trials (OBA effect). Because we were interested in how spatial cue reliability would modulate these attention effects, we also computed linear mixed models to test for significant differences in the magnitude of these attention effects between groups. The models included spatial-validity condition, target location (restricted to valid/same-object for SBA, and same-object/different-object for OBA), and their interaction as fixed effects, with random intercepts for subjects. These models were then used to test for differences in the magnitude of attention effects and RTs for each target location between the high and low spatial-validity groups.

### Pupil diameter analyses: Attention effects

Our primary question surrounded how trial-level changes in pupil diameter would relate to indices of attentional modulation. In particular, we sought to determine whether phasic LC-NE activity modulates SBA and OBA effects, where such effects are defined as a change in performance between two trial types. Recognizing that much of the variance in attentional state may occur within participants (Esterman et al., 2013; Hopstaken et al., 2015; Mittner et al., 2016; Posner & Rafal, 1987), especially over several multi-hour testing sessions, we elected to focus on trial-level analyses over participant-level analyses that may obscure such trial-by-trial variability (see Online Supplemental Materials (OSM) for participant-level analyses and results). We thus trichotomized the data according to pupil diameter and examined attention effects within the two extreme groups. This approach results in a loss of data from trials with intermediate pupillary responses, but allows for a clear separation between groups, such that neighboring pupillary responses are not classified as qualitatively distinct because they happen to fall on either side of the midline. We suspected that each participant likely experienced periods of both high and low attentional focus throughout the multi-day testing sessions (Esterman et al., 2013; Hopstaken et al., 2015; Mittner et al., 2016; Posner & Rafal, 1987). However, the extent to which a single participant’s focus reaches and remains in either extreme may vary on an individual basis. Because trichotomizing trials within participant would force data from each individual into all three pupil size groups regardless of range, we instead elected to sort trials according to whether the change in pupil diameter following cue presentation was above the 66th (large phasic response) or below the 33rd (small phasic response) percentile across all participants. This approach allows for the possibility that some individual participants may have more or less stable attentional states than others.

The attentional prioritization hypothesis predicts that participants will deploy attention to the display in a controlled manner to optimize task performance, and accordingly implicates flexible, top-down attention (Shomstein, 2012). Thus, it is possible that between-group differences in OBA effects will only emerge when participants are in a focused attentional state, such that they actively exert top-down control to guide their search. Conversely, periods of low attentional focus would be expected to diminish possible group differences in OBA that are driven by the proportion of invalid trials, because participants may not exert the top-down control required to select object contours to optimally prioritize probable target locations.

#### Pupillometry preprocessing

Saccades were computed automatically using the standard eye movement and velocity thresholds in the EyeLink software package. Offline, trials that contained saccades and blinks were removed from further analyses.

Pupil area measurements were first extracted from the eye-tracker in 100-ms bins (50 samples per bin) and then converted to diameter using $2\sqrt{area/\pi }$ before analyses. The mean pupil diameter within each bin was then normalized to each participant’s within-session mean (z-scored within session) to account for potential participant- and session-level variability in the mean and variance of the pupil response. Visual inspection of diagnostic plots revealed that pupil diameter measurements were approximately normally distributed, so no further transformations were performed.

Baseline pupil diameter estimates were then calculated as the average pupil diameter during the first 100 ms of each trial (time-locked to pre-cue display presentation). Pupil diameter at target presentation was estimated by calculating the mean in the 100-ms bin that contained the onset of the target. For example, the trial period between 500 and 600 ms was used to estimate terminal pupil diameter across all trials with target onsets at 500, 525, 550, and 575 ms. Given previous measurements of an approximately 100-ms latency between stimulus onset and the LC phasic response (Aston-Jones et al., 1994; Aston-Jones & Cohen, 2005), we do not expect the small temporal deviations in our time-locking procedure to impact the results. Finally, our measure of phasic pupil response was calculated by subtracting the normalized terminal pupil diameter (at target onset) from the normalized baseline pupil diameter (at cue onset), consistent with current recommendations (Mathôt et al., 2018) and several recent empirical studies (Eldar et al., 2013; Gilzenrat et al., 2010; Laeng et al., 2011; Mathôt et al., 2014).

#### Statistical analyses

The pupil diameter grouping factors described above were used to test the prediction that phasic LC-NE activity, and correspondingly, pupil diameter (Aston-Jones & Cohen, 2005; Costa & Rudebeck, 2016; Joshi et al., 2016; Rajkowski et al., 1994; Reimer et al., 2016), may track how participants distribute attention during the task (Alnæs et al., 2014; Aston-Jones et al., 1994; Gabay et al., 2011; Eldar et al., 2013; Eldar et al., 2016; Gilzenrat et al., 2010; Mittner et al., 2016; Thiele & Bellgrove, 2018). All statistical tests examining attention effects in RT as a function of the pupillary response were restricted to correct trials only. We first examined the effects of pupil diameter across all task conditions by computing a linear mixed model including all predictor variables (spatial validity condition, target location, pupil diameter, and their interactions) modeled as fixed effects and random intercepts for subjects. To address the primary question of the study, we tested whether the magnitude of SBA and OBA effects significantly differed between the high and low spatial-validity groups as a function of the pupillary response, using mixed models with fixed effects for spatial validity condition, target location, pupil diameter, and their interactions (restricted to valid/same-object target locations for SBA, and same-object/different-object target locations for OBA). In these cases, the presence of a significant three-way interaction effect would indicate that the size of SBA and/or OBA effects were differentially modulated by pupil diameter between spatial-validity groups.

Following the between-group tests, planned comparisons testing for individual attention effects (SBA and OBA) were carried out using paired t-tests within each subset of conditions (large vs. small pupil diameter within high and low spatial-validity groups). To test for significant within-group differences in the size of SBA and OBA effects associated with the pupillary response, we performed linear mixed models with random intercepts for subjects and fixed effect variables for pupil size, target location, and their interaction (with targets restricted to valid/same-object for SBA, and same-object/different-object for OBA).

### Pupil diameter analyses: Control measures

#### Assessing the relationship between pupil size and performance

Before trichotomizing the data based on pupil size (see *Pupil diameter analyses: Attention effects* above), we first checked that our expectation regarding pupil diameter and behavioral performance was met. First, we examined the Pearson correlations between trial-level pupillary responses and trial-level RTs within each group. Here, we predicted trial-level pupil diameter should be negatively correlated with trial-level RT, such that larger phasic responses tend to be associated with faster RTs in both spatial-validity groups (Aston-Jones & Cohen, 2005; Gelbard-Sagiv et al., 2018). Moreover, we performed a two-sample t-test between groups (unequal variances) to ensure that the size of the mean pupillary response – and by extension, the degree of attentional control – was well matched on average across spatial validity conditions.

#### Trial counts across subjects and conditions

After the trichotomization procedure, we checked whether each participant contributed enough trials to be included in the analyses, and that the proportion of both large and small pupil trials were well-matched between spatial-validity groups. Trial counts were conducted after excluding incorrect trials and those containing blinks or saccades.

Because our procedure employed a variable delay period with cue-to-target ISIs ranging from 300 to 1,175 ms, we anticipated that temporal lag in the pupillary response may affect the proportion of trials from different delays contributing to the small versus large pupil diameter classifications. Specifically, trials with longer ISIs may be more likely to capture the peak of the pupillary response to the cue, resulting in a greater proportion of these trials contributing to the large pupil condition versus the small pupil condition. To ensure that this possibility did not unduly influence our results, we coarsely divided trial ISIs into early (300–575 ms), intermediate (600–875 ms), and late (900–1,175 ms) delays to examine how they were distributed across pupil diameter groups for each spatial validity condition.

#### Checking for new confounds

It was critical to establish that new confounds were not inadvertently introduced by categorizing pupillary responses by size. Thus, we sought to establish that, after classifying trials according to pupil diameter, (1) the size of the pupillary response was matched across task conditions for each pupil group (small and large), and (2) target contrast did not differ between pupil groups (see *Titration procedure* above). The former ensures that any performance differences between cue types cannot be attributed to differences in pupil diameter, while the latter ensures that pupil diameter can be attributed to the observer’s internal attentional state and not physical display properties. To test these assumptions, we computed two linear mixed models with raw (pre-normalized) pupil diameter and target contrast as the respective outcome variables, and fixed effects for pupil diameter group (small/large), spatial validity condition, and target location, including their interactions.

### Effect-size calculations

To elucidate the size and strength of the reported effects, we include Cohen’s *d* for all t-tests using the formula:

$$ \left({M}_1\hbox{--} {M}_2\right)/{SD}_{pooled} $$

where values of 0.2, 0.5, and 0.8 roughly indicate small, medium, and large effect sizes, respectfully. A Bayes Factor analysis is also included for all statistical comparisons – allowing for a direct comparison between the alternative and null hypotheses (Rouder et al., 2009; Wagenmakers, 2007) – in addition to traditional p-values. Bayes Factors (BF_10_) were obtained using the Bayesian information criterion (BIC) approximation method (Wagenmakers, 2007), such that larger values indicate greater probabilistic evidence for the alternative hypothesis relative to the null hypothesis.

## Results

### Behavioral results

**Fig. 3:**
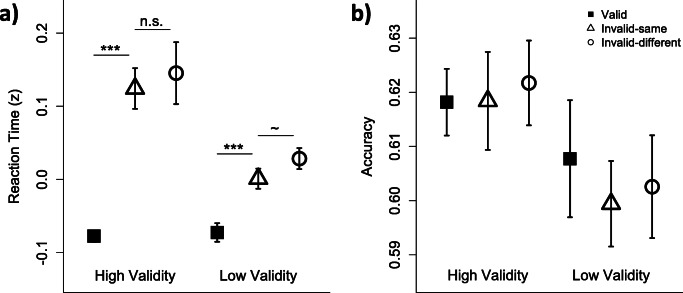
Behavioral results. **(a)** Reaction time and **(b)** accuracy for high (left) and low (right) spatial-validity groups, for valid (filled square), invalid-same (open triangle), and invalid-different (open circles) trials. Error bars depict between-subject SEMs. ‘***’ = p < .001. ‘~’ = marginally significant (p = .06). For accuracy, all ps > .05

Mean response accuracy was above chance (*M* = 61.1%, *SD* = 3.5%), and did not significantly differ between high (*M* = 61.9%, *SD* = 2.6%) and low (*M* = 60.4%, *SD* = 4.2%) spatial cue validity groups, *t* (34) = 1.21, *p* = 0.23, *d* = 0.42, BF_10_ = 0.36. This is in line with our staircasing procedure (see *Methods*), which also produced similar contrast estimates between groups to reach criterion performance (high validity: *M* = 31% contrast, *SD* = 16%; low validity: *M* = 38% contrast, *SD* = 20%), *t* (34) = 1.24, p = 0.23, *d* = 0.42, BF_10_ = 0.37. Likewise, mean RTs were well matched between the high (*M* = 538 ms, *SD* = 125 ms) and low (*M* = 530 ms, *SD* = 107 ms) spatial cue validity groups, *t* (34) = 0.21, *p* = 0.83, *d* = 0.07, BF_10_ = 0.17; see Fig. S1, OSM. Although we first looked for attention effects in both accuracy and RT before embarking on other analyses (see Fig. 3), they were uniformly absent in accuracy. The following analyses thus pertain exclusively to effects in RT on correct trials, which is consistent with a large body of OBA studies (e.g., Egly et al., 1994; Moore et al., 1998; Shomstein & Yantis 2002, 2004; Watson & Kramer, 1999). Notably, we verified that the observed results described below were not the product of a speed-accuracy trade-off: Pearson correlations between RT and accuracy were negative and significant on average, suggesting that correct responses were associated with faster RTs in our data, *r* = -0.04, 95% confidence interval (CI) [-0.06, -0.02], *t* (35) = -4.80, *p* < 0.001.

A linear mixed model tested for group effects of spatial-validity condition (high and low validity), target location (valid, invalid same-object, and invalid different-object), and their interaction (see Fig. 3a). The model revealed a significant interaction between target location and spatial-validity condition, *F* (2, 68) = 8.34, *p* < 0.001, BF_10_ = 24.83. Although both groups exhibited significant SBA effects in RT (high-validity: *M*_*diff*_ = 0.20, *t* (16) = 6.64, *p* < 0.001, *d* = 2.38, BF_10_ > 100; low-validity: *M*_*diff*_ = 0.07, *t* (18) = 5.19, *p* < 0.001, *d* = 1.26, BF_10_ > 100), the effect was reliably larger in the high-validity group, *F* (1, 34) = 15.7, *p* < 0.001, BF_10_ > 100. Evidence for attentional prioritization was apparent when we considered the invalid trials alone: unequal variance t-tests revealed that RTs on invalid trials were significantly faster for the low validity group compared with the high validity group, *M*_*diff*_ = 0.12, *t* (20) = 3.31, *p* = 0.003, *d* = 1.15, BF_10_ = 37.26, and no group differences were observed for valid RTs, *M*_*diff*_ = 0.01, *t* (30) = -0.33, *p* = 0.74, *d* = 0.11, BF_10_ = 0.18. Thus, participants appeared to prioritize likely target locations, as predicted based on previous findings (Shomstein & Behrmann, 2008; Shomstein & Yantis, 2004).

No OBA effect was detected for the high-validity group, *M*_*diff*_ = 0.02, *t* (16) = 0.99, *p* = 0.34, *d* = 0.14, BF_10_ = 0.28, while a medium-sized OBA effect in the low-validity group was marginally significant, *M*_*diff*_ = 0.03, *t* (18) = 2.02, *p* = 0.06, *d* = 0.44, BF_10_ = 1.13. However, the patterns across groups did not significantly differ, *F* (1, 34) = 0.07, *p* = 0.80, BF_10_ = 0.12. These results suggest that, while OBA effects may have been weakly present for a preponderance of low spatial-validity participants, object contours were unlikely the exclusive driving force of attention effects across the board.

### Pupil diameter results: Control measures

Before testing the prediction that reliable OBA effects may only emerge during periods of high attentional focus, we first describe the outcome of our control measures to verify the appropriateness of our novel analysis approach.

#### Assessing the relationship between pupil size and performance

Trial-by-trial pupillary responses weakly but reliably predicted trial-by-trial RT for both groups (high validity condition: mean *r* = -0.04, 95% CI [-0.06, -0.02], *t* (16) = -4.79, *p* < 0.001, BF_10_ > 100; low validity condition: mean *r* = -0.02 95% CI [-0.05, -0.01], *t* (18) = -2.63, *p* = 0.02, BF_10_ = 5.01). This trial-level relationship is consistent with the known relationship between phasic norepinephrine release and task performance (Aston-Jones & Cohen, 2005; Gelbard-Sagiv et al., 2018). Furthermore, an unequal variances two-sample t-test revealed no significant differences in the mean pupillary response between high (*M* = 0.13 units, *SD* = 0.33) and low (*M* = 0.25 units, *SD* = 0.54) spatial-validity groups, *t* (31) = -0.78, *p* = 0.44, *d* = 0.27, BF_10_ = 0.23. Together, these results indicate that our expectations about the directional relationship between pupil size and task engagement holds, and that both groups voluntarily deployed attention to an equivalent degree.

#### Trial counts across subjects and conditions

After trichotomization, all participants were represented in both the large and the small pupillary response groups, with a similar total number of trials falling above the 66th percentile (high validity: 11,454; low validity: 11,371) and below the 33rd percentile (high validity: 11,936; low validity: 12,697). Critically, each participant contributed a sufficient number of correct trials to each of the cells included in the analysis (*M* = 661 trials per subject, range = 209–1,312).

Given the variable ISIs in our design, it is possible trials with the longer delays were associated with larger peak pupil diameters at target onset due to temporal lag in the pupillary response. In light of this expected relationship, we examined the number of trials drawn from early (300–575 ms), intermediate (600–875 ms), and late (900–1,175 ms) delays to establish that the proportion of trials at each ISI sorted into large and small pupil diameter groups were matched across the two spatial-validity groups. The number of trials drawn from each delay with small relative pupil diameters did not follow a monotonic pattern (early: 7,547 trials; intermediate: 9,130 trials; late: 7,956 trials), but notably, an ample number of trials from each delay period contributed to the analysis. Consistent with a temporal lag in the peak of phasic responses, relatively large pupil diameters were more frequently observed on trials with longer ISIs (early: 5,759 trials; intermediate: 7,976 trials; late: 11,279 trials). From our perspective, the presence of this expected association does not pose an issue for subsequent analyses, provided that the pattern is matched across experimental conditions. We confirmed that the same relationships between ISI and pupil diameter frequency were expressed in both the high and low spatial validity conditions, χ^2^ (2) = 3.4, *p* = 0.18 (see Table S1, OSM).

#### Checking for new confounds

We next examined the data to ensure that, after sorting and classifying trials based on pupil size, we did not inadvertently introduce critical confounds.

**Fig. 4:**
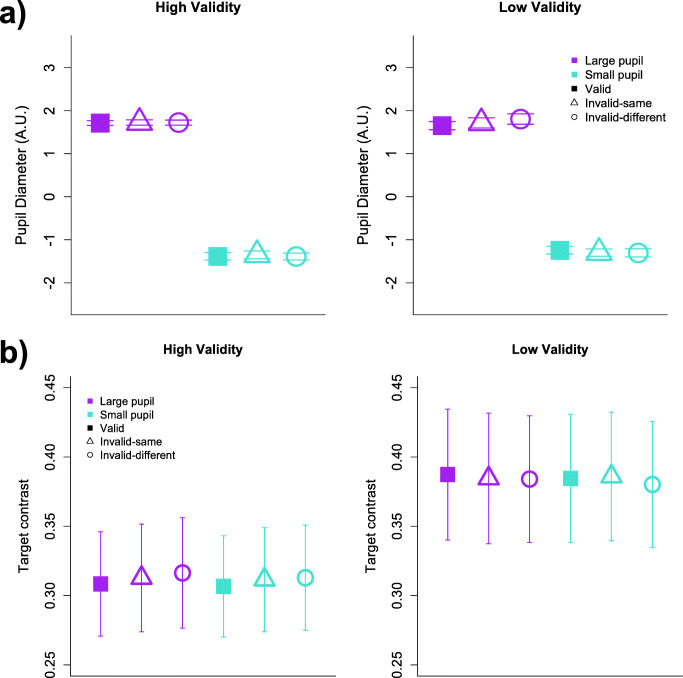
Control measures. **(a)** Raw pupillary response across task conditions in arbitrary units. Mean change in pupil diameter differs according to trial grouping (above the 66th and below the 33rd percentiles), but is matched with respect to spatial-validity conditions and target location. **(b)** Percent target contrast across task conditions. No contrast differences were observed across spatial-validity conditions, target locations, or trial groupings related to the size of the pupillary response (all ps > .05). Error bars depict between-subject SEMs

As expected, mean raw pupil size differed significantly between large and small pupillary response groups (*F* (1, 34) = 1,350, *p* < 0.001, BF_10_ > 100), but importantly, it was well matched across spatial validity conditions (*F* (1, 34) = 0.08, *p* = 0.78, BF_10_ = 0.06) and trial types within pupillary response groups (*F* (2, 136) = 1.25, *p* = 0.29, BF_10_ < 0.001; see Fig. 4a). Similarly, target contrast was matched across pupillary response groups (*F* (1, 34) = 0.40, *p* = 0.53, BF_10_ = 0.001), spatial validity conditions (*F* (1, 34) = 1.45, *p* = 0.24, BF_10_ = 0.02), and target locations (*F* (2, 136) = 0.34, *p* = 0.71, BF_10_ < 0.001; see Fig. 4b). These results are consistent with the expectation that measures of the pupillary response taken during the cue-to-target interval should be unaffected by properties of the stimulus display, and instead should reflect internal changes in attentional control state.

### Pupil diameter results: Attention effects

Our behavioral results revealed several differences in attention effects across spatial-validity groups, consistent with attentional prioritization. In particular, decreasing the reliability of an endogenous pre-cue led to smaller SBA effects, coupled with faster RTs to invalid targets and a marginally significant OBA effect that was not observed in the high spatial-validity group. However, a key prediction of the prioritization account is that such shifts in attention require flexible, top-down attention. If this is the case, attention effects driven by top-down processing may be overshadowed in our behavioral results if participants experienced fluctuations in task engagement and attentional focus over the course of the 1.5-h testing sessions (Esterman et al., 2013; Hopstaken et al., 2015; Mittner et al., 2016; Posner & Rafal, 1987). To test this possibility, we used pupil diameter as a physiological index of top-down attention (Eldar et al., 2013; Eldar et al., 2016; Gabay et al., 2011; Geva et al., 2013; Gilzenrat et al., 2010; Laeng et al., 2011; Mittner et al., 2016; Rondeel et al., 2015; Wendt et al., 2014), under the assumption that the effects of the spatial validity manipulation would be more pronounced when pupil diameter and associated LC-NE activity were relatively elevated.

**Fig. 5:**
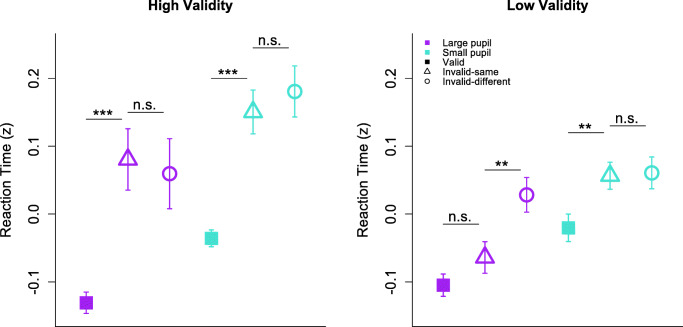
Participant-level effects of pupillary response (above the 66th and below the 33rd percentiles), spatial-validity condition, and target location on reaction time. Error bars depict between-subject SEMs

Accordingly, we computed a linear mixed-effects model to examine how large and small pupillary responses interacted with cue validity for both the high and low spatial-validity groups (Fig. 5). Consistent with the trial-level correlations that indicated an RT benefit associated with larger pupil diameters, we observed a significant main effect of pupillary response magnitude, *F* (1, 34) = 24.7, *p* < .001, BF_10_ > 100. Across both spatial-validity groups, RTs were faster on trials where participants’ pupils showed larger relative increases in diameter before target onset. The RT benefit of larger phasic pupillary responses in our study is consistent with previous work (Geva et al., 2013; Gilzenrat et al., 2010; Hopstaken et al., 2015; Laeng et al., 2011; Rondeel et al., 2015; Wendt et al., 2014), and further suggests that our PD measure was reliably associated with fluctuations in attentional focus.

Critically, we predicted that object-based effects associated with the probability manipulation would be contingent on top-down attention, such that group differences in OBA are more pronounced when pupil diameter (and by proxy, LC-NE activity) is relatively high. Our results supported this prediction: restricting the mixed-effects model described above to the relevant comparison conditions for OBA effects (same- and different-object invalid target locations), we found a significant three-way interaction between target location, spatial validity condition, and pupil diameter, *F* (1, 68) = 4.92, *p* = 0.03, BF_10_ = 1.03. Specifically, this interaction reveals that OBA effects significantly differed between spatial-validity groups only when the pupillary response was relatively large, consistent with a role of top-down control in attentional prioritization (see Fig. 5).

Conversely, when the model was restricted to target locations testing for SBA effects (valid and same-object invalid locations), the three-way interaction between target location, spatial validity condition, and pupil diameter was not significant, *F* (1, 68) = 0.93, *p* = 0.34, BF_10_ = 0.14. Thus, in contrast to object-based effects, differences in the magnitude of SBA effects between probability conditions do not appear to be driven by pupillary responses.

To further clarify how the attention effects in our study were modulated by pupil diameter, we next examined within-group effects of pupillary response magnitude on SBA and OBA. Within the high spatial-validity group, paired t-tests revealed significant SBA effects for both large (*M*_*diff*_ = .211, *t* (16) = 4.34, *p* < .001, *d* = 1.5, BF_10_ > 100) and small (*M*_*diff*_ = .186, *t* (16) = 6.07, *p* < .001, *d* = 1.8, BF_10_ > 100) pupillary responses, and these effects did not differ in magnitude (*F* (1, 32) = .19, *p* = .67, BF_10_ = 0.13, see Fig. 5a). OBA effects were equally absent in the high-validity condition regardless of the size of pupillary responses (large: *M*_*diff*_ = .021, *t* (16) = -.50, *p* = .63 *d* = .11, BF_10_ = 0.20; small: *M*_*diff*_ = .030, *t* (16) = .86, *p* = .40 *d* = .21, BF_10_ = 0.25), *F* (1, 32) = .87, *p* = .36, BF_10_ = 0.18. Thus, apart from a main effect of speeding RTs to all possible target locations, the size of the pupillary response did not significantly affect how attention was distributed across the display when the reliability of a spatial cue was high.

Conversely, changes in pupil diameter differentially modulated the size of attention effects in the low spatial-validity condition, providing strong support for the predictions of the attentional prioritization account. We observed a reliably large OBA effect for trials with a large pupillary response (*M*_*diff*_ = .092, *t* (18) = 3.47, *p* = .003, *d* = .86, BF_10_ = 21.10) but not trials with a small response (*M*_*diff*_ = .004, *t* (18) = .21, *p* = .84, *d* = .04, BF_10_ = 0.17). A linear mixed-effects model revealed a significant difference in slopes between OBA effects for large and small pupillary response trials (*F* (1, 36) = 7.00, *p* = .01, BF_10_ = 3.35).

Together, these results suggest that the magnitude of OBA effects were modulated by LC-NE activity in the low spatial-validity condition but not in the high spatial-validity condition.^1^ Our between-group analyses revealed that the size of OBA effects on trials with a large phasic response were significantly different between spatial validity conditions, despite no reliable difference in target contrast or in the pupil sizes themselves (see Fig. 4). Much like the behavioral findings, this pattern is most consistent with a prioritization account of object-mediated space-based selection. Moreover, by showing that OBA effects emerge specifically when phasic pupillary responses are large, our results provide critical physiological evidence implicating top-down attention as a precursor to object-based effects.

## Discussion

In the present study, we investigated whether object-based spatial selection is under voluntary control during a modified two-rectangle paradigm. Following the logic of the attentional prioritization account, we manipulated the reliability of the endogenous pre-cue so that it was either 80% accurate (high spatial-validity group) or 50% accurate (low spatial-validity group) to in turn predictably shift the behavioral likelihood of attending to the object contours. Both groups effectively utilized the spatial cue, as indicated by significant SBA effects. Consistent with an attentional probability matching approach, the high spatial-validity group exhibited a significantly stronger effect. Similarly, the low spatial-validity group responded significantly faster to the (relatively more probable) invalidly cued targets compared to the high spatial-validity group, while also exhibiting a marginal OBA effect. Together, the behavioral patterns point to an attentional prioritization of probable target locations in support of behavioral goals, which may supersede object-mediated spatial selection (Shomstein & Yantis, 2002, 2004).

Shomstein and Behrmann (2008) showed OBA effects emerge on top of probabilistic ones when physical display parameters elicit strong object-based representations, such as by (1) providing sufficient encoding time and/or (2) rendering objects in different colors, while Shomstein (2012) argues that object-based selection is, in part, a flexible process. In line with this proposal, we show OBA effects can emerge even in unmanipulated displays with fluctuations in top-down attention, as indicated by phasic pupillary responses. We extracted pupil diameter during the cue-to-target interval, in which all stimulus parameters were matched across within-subject manipulations of trial-by-trial cue validity and between-subject manipulations of cue reliability. Despite no changes in external stimulation, we observed large differences in the pupillary response across trials that we therefore attribute to internal cognitive mechanisms (Eldar et al., 2016; Mather et al., 2016). Here, we interpret a relatively large transient pupillary response to the pre-cue display as indicative of participants exerting greater attentional focus. This expectation culled from the literature (e.g., Alnæs et al., 2014; Gabay et al., 2011; Geva et al., 2013; Gilzenrat et al., 2010; Thiele & Bellgrove, 2018) is further supported in our data: the response to the cue display was predictably associated with RT, thus serving as a good index of top-down attention in the current task.

Given that a predictive endogenous pre-cue was used in both probability conditions, SBA was uniformly expected to be under voluntary control. We reasoned that if OBA was similarly controlled, then attentional focus – and by extension, pupil diameter – should be relatively high in cases where object selection occurs. We furthermore reasoned that any relationship between OBA and pupil diameter should depend on the behavioral expediency of object selection. If object-based spatial selection is instead the result of an automatic attentional spread, then the size of OBA effects and any relationship they have with the pupillary response should not depend on cue reliability.

The pupillometry results were most consistent with voluntarily controlled attentional prioritization. Trials with relatively high within-subject cue-locked responses were associated with a significant OBA effect for only the low spatial-validity condition – where invalid locations were more likely to contain a target than the high spatial-validity condition. An equally large response in the high spatial-validity condition resulted in speeded responses to the target across all locations, while the magnitude of a pure SBA effect was matched across trials with a small phasic response.

A priori, one might have expected to see a significantly greater SBA effect on large pupillary response trials compared to their small counterparts in the high spatial-validity group, under the assumption that more attentional effort may be needed to both selectively facilitate performance at the spatially cued location and suppress an attentional spread to the invalid-same object location. To the contrary, however, our results demonstrated uniform facilitation across target locations without a concurrent redistribution of attention. This may suggest that additional effort was not needed to suppress object contours. Rather, when considered in conjunction with the pattern observed in the low spatial-validity condition, our results suggest that cognitive demands were greater when attending to object contours. This might explain why behavioral evidence for object-based selection only emerged when this approach was behaviorally expedient.

Several studies have demonstrated that LC-NE activity magnifies representational selectivity (Eldar et al., 2013; Eldar et al., 2016) through interactions with glutamate (Mather et al., 2016). Eldar and colleagues argue that when NE-mediated gain is high, the most salient stimuli are afforded a representational boost while representations of non-salient stimuli are suppressed, where salience is driven by a combination of top-down and bottom-up factors. The pattern of results reported here suggest that the physical properties of the object contours were not more salient than the endogenous spatial cue and associated target location probabilities: when gain was relatively high (as reflected by pupil size; see Fig. 4) for the high spatial-validity group, only a space-based attention effect was observed. In contrast, high gain of the same magnitude within the low spatial-validity group corresponded to an object-based attention effect. Given these differing patterns between groups despite matched external stimuli and pupil size, we conclude that object contour saliency – leading to an OBA effect – must have been internally elevated, driven by the behavioral expediency of object-based selection when target position certainty was low.

Because we used pupil size as an indirect assay for LC-NE activity, we cannot rule out other mechanisms that could be contributing to the pattern of results described here. For example, in an electrophysiological study with nonhuman primates, Joshi et al. (2016) found that, among all tested sites, neuronal activity within the LC best predicted temporally precise changes in pupil size, whether they were spontaneous or event-driven. However, significant effects of smaller magnitudes were also found within the superior and inferior colliculi and cingulate cortex. Notably, such relationships may reflect broad coordination across areas that originated from the LC, given interconnectivity between regions. Still, various studies have linked NE, dopamine (DA), and acetylcholine (ACh) to changes in pupil size (Hauser et al., 2019; Reimer et al., 2016; Varazzani et al., 2015), leading to attempts to dissociate their relative contributions. Relevant to the current study, transient pupil dilations were shown to be correlated with the NE system, while sustained dilations were associated with ACh activity in mouse models (Reimer et al., 2016), and pupil size during the pre-cue period of an effortful task significantly correlated with activity from LC neurons but not DA-producing substantia nigra neurons in rhesus monkeys (Varazzani et al., 2015). Regardless of the underlying physiological source, though, even more studies have linked changes in pupil size to attentional or cognitive control (Einhäuser et al., 2008; Eldar et al., 2013; Eldar et al., 2016; Gabay et al., 2011; Geva et al., 2013; Gilzenrat et al., 2010; Hopstaken et al., 2015; Laeng et al., 2011; Rondeel et al., 2015; Wendt et al., 2014). Thus, any mechanistic debate should not detract from the larger argument presented here regarding top-down control of object-mediated space-based selection.

Together, the behavioral and pupillometry results converge on a voluntary prioritization account of object-based spatial selection, while at the same time producing no evidence of an automatic spread of attention. Because our design manipulated spatial selection via use of a predictive endogenous cue, our results may only be specific to voluntarily guided attention. For example, several studies have reported significant OBA effects during a two-rectangle paradigm when paired with a peripheral, exogenous cue but not with central, endogenous cues (Arrington et al., 2000; Lauwereyns; 1998; Macquistan, 1997). Importantly though, this is not a steadfast dichotomous relationship, as others have found significant OBA effects using endogenous cues (Abrams & Law, 2000; Chen & Cave, 2008; Goldsmith & Yeari, 2003). Furthermore, while many two-rectangle paradigm studies utilize an exogenous peripheral cue, they are often concurrently reliable and paired with long ISIs (Egly et al., 1994; Fiebelkorn et al., 2013; Shomstein & Yantis, 2004). This makes it challenging to disentangle the contributions of voluntary and involuntary attention mechanisms to observed OBA effects. It would thus be worthwhile to see if a similar relationship between attention effects and pupil size would emerge with an uninformative exogenous cue and consistently short delay period.

An important component of the attentional prioritization account is that only probable target locations are strategically selected. With sufficient top-down control, the invalid, same-object location is afforded preferential processing over the invalid, different-object location via gestalt mechanisms that group it with the cued location, allowing for an efficient search for the behaviorally relevant target (Shomstein, 2012). However, intervening locations within the same object are not afforded an attentional benefit. Although other studies have provided support for this component of the account (e.g., Shomstein & Yantis, 2002), we cannot rule out the possibility that the whole rectangle was selected when our participants adopted an object-based strategy. Thus, the larger response to the display in the low probability condition could be indicative of whole object processing. Adding an incompatible intervening distractor to our current task while still employing some measure of LC-NE activity could reveal whether OBA effects derived during a relatively focused attentional state results in full object selection, or discrete selection of probable target locations grouped by a common region. Importantly, though, our results indicate that such object-based selection should largely be driven by task demands.

The current study adds to a line of research indicating that object-based spatial selection is not obligatory. Here, OBA appears to be voluntarily deployed according to behavioral context: we only find such behavioral effects when the likelihood of a target appearing in the invalid, same-object location is sufficiently high. Furthermore, we used cue-locked pupil diameter to noninvasively measure phasic LC-NE activity, motivated by numerous studies indicating that relative activation of the LC-NE system correlates with attentional effort (Alnæs et al., 2014) and affects processing of behaviorally relevant stimuli (Aston-Jones & Cohen, 2005; Gabay et al., 2011; Gelbard-Sagiv et al., 2018). Via pupillometry, we demonstrated that when shifting attention across a display is advantageous, object-based effects are supported by a relatively focused attentional state. These results are the first to our knowledge to demonstrate that the LC-NE system not only affects visual target processing, but also tracks attentional strategy.

## Supplementary Information

ESM 1 (PDF 256 kb)

## Data Availability

All behavioral and eye-tracking data reported in this paper, as well as custom R code used for analyses, are available on the Open Science Framework (https://osf.io/48thk/)

## Footnotes

1 Critically, we verified that including intermediate response trials by performing a median split along pupil size did not qualitatively change the results (except for a slightly stronger SBA effect in the low validity, large phasic-response group).
