# Supplementary material for: Phasic pupillary responses modulate object-based attentional prioritization
Source: Atten Percept Psychophys. 2021 Jan 27;83(4):1491–507. doi: 10.3758/s13414-020-02232-7 (PMC8084782; doi:10.3758/s13414-020-02232-7)
Supplement: Supplementary file 1 — (PDF 256 kb) [file 13414_2020_2232_MOESM1_ESM.pdf]

Supplemental Material

Phasic pupillary responses modulate object-based attentional prioritization

Participant-level relationship between pupil diameter and attention effects. In addition to our primary analysis examining how trial-level changes in pupil diameter may modulate attention indices across spatial validity conditions, we examined whether the mean pupillary response was associated with different attentional prioritization patterns at the participant level. To do so, individual participant means were calculated and then correlated with mean RTs (across all trial types), OBA effects, and SBA effects across all cue-to-target ISIs.

Pearson correlations between participant-level mean RTs and the mean cue-to-target pupillary response revealed that mean change in pupil diameter was not significantly associated with overall task RTs in the high ($r = -0.26$, 95% CI [-0.66, 0.25], $t\,(15) = -1.04$, $p = 0.31$) or low validity conditions ($r = -0.20$, 95% CI [-0.60, 0.28], $t\,(17) = -0.83$, $p = 0.42$). Similarly, mean change in pupil diameter in the high validity condition did not significantly correlate with the magnitude of SBA ($r = 0.17$, 95% CI [-0.34, 0.60], $t\,(15) = 0.68$, $p = 0.51$) or OBA effects in RT ($r = 0.37$, 95% CI [-0.13, 0.72], $t\,(15) = 1.56$, $p = 0.14$). This was true for the low validity group as well (SBA effects: $r = 0.33$, 95% CI [-0.15, 0.68], $t\,(17) = 1.44$, $p = 0.17$; OBA effects: $r = -0.41$, 95% CI [-0.73, 0.06], $t\,(17) = -1.84$, $p = 0.08$).

The associations between phasic pupillary responses and attention effects at the participant level are largely weak or absent. One possibility for this is that much of the variance in attentional state may occur within participants (Esterman et al., 2013; Hopstaken et al., 2015; Mittner et al., 2016; Posner & Rafal, 1987), and trial-by-trial variability is lost when we calculate individual participant means. This point is illustrated when we consider participant-level and

trial-level correlations collapsed across trial types: mean change in pupil diameter did not correlate with overall task RT at the participant level for either spatial validity condition, but trial-by-trial pupillary responses exerted a consistent, albeit small effect on RT (see Results, main text).

We interpret our results from the low spatial validity condition in terms of voluntary control over object-based spatial selection. Notably, though, our participant-level analyses suggest that this attentional strategy was not sustained for all participants over the course of the multiple experimental sessions— apart from a marginally negative association with the OBA effect in the low validity group, no significant participant-level correlations between mean pupil diameter and attention effects were present in our data. Importantly, this negative relationship effectively reversed when we accounted for trial-level variability in our analyses: then only comparatively large pupil diameters were associated with significant OBA effects. This suggests that, in cases of dynamic attentional deployment, relying solely on participant means may lead to incomplete or even inappropriate conclusions. In the same vein, we did not observe a reliable relationship between pupil diameter and overall task RT when both were averaged within participant. However, these patterns emerged strongly when we accounted for trial-level variability in our analyses. This may be in line with other studies that have demonstrated fluctuations in cognitive effort over time with monotonous tasks (Gilzenrat et al., 2010; Langner & Eickoff, 2013; Mittner et al., 2016). Our findings illustrate the utility of examining data at the trial level, especially during extended selective attention tasks. Furthermore, measuring pupil diameter during such tasks may allow researchers to make more nuanced predictions about the expected presence and size of attention effects.

Supplementary Figure 1.

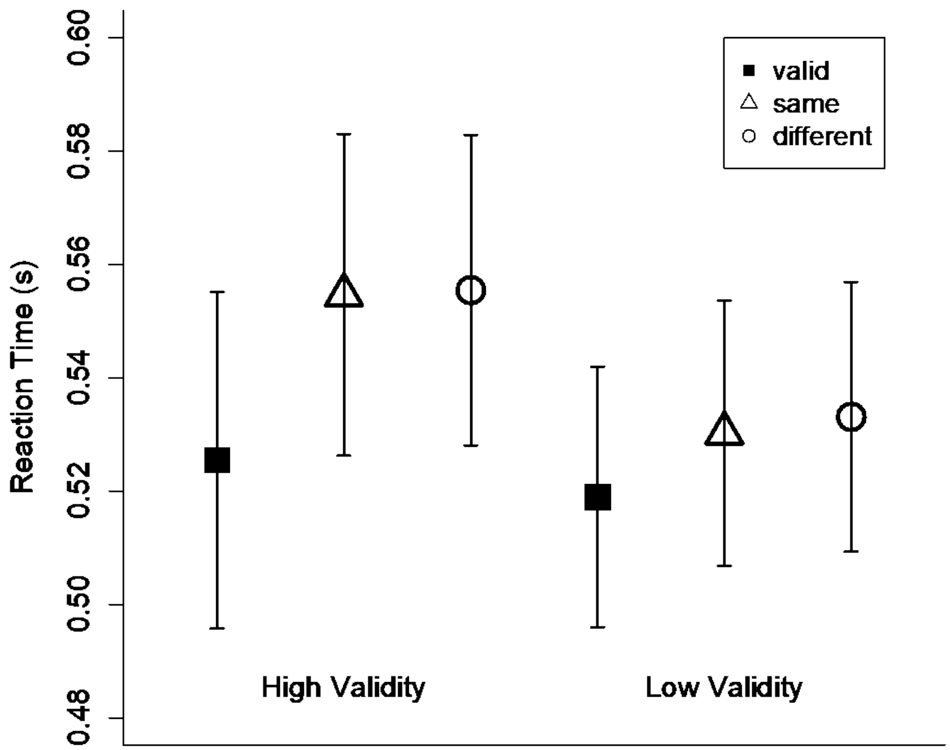

Figure S1. Behavioral results in untransformed RT for high (left) and low (right) spatial validity groups, across valid (filled square), invalid-same (open triangle) and invalid-different (open circles) trials. Error bars depict between-subject SEM. Note that the results reported in the main text were z-scored within session to control for subject- and session-level variability in mean RT.

Supplementary Table 1.

	**High Spatial Validity**		**Low Spatial Validity**	
	**Small PD**	**Large PD**	**Small PD**	**Large PD**
**300 – 575 ms**	3,687	2,822	3,860	2,937
**600 – 875 ms**	4,497	3,914	4,633	4,062
**900 – 1175 ms**	3,752	5,665	4,204	5,614

Table S1. Trial numbers contributing to the pupil diameter and attention effect analysis across spatial validity conditions, pupil size categories, and trial ISIs. Variable ISIs are divided into early (300 – 575 ms), intermediate (600 – 875 ms), and late (900 – 1175) delay categories.

References

Esterman, M., Noonan, S.K., Rosenberg, M. & DeGutis, J. (2013). In the zone or zoning out? Tracking behavioral and neural fluctuations during sustained attention. *Cereb Cortex* **23**, 2712–2723.

Gilzenrat, M.S., Nieuwenhuis, S., Jepma, M., & Cohen, J.D. (2010). Pupil diameter tracks changes in control state predicted by the adaptive gain theory of locus coeruleus function. *Cognitive, Affective, & Behavioral Neuroscience* **10,** 252–269.

Hopstaken, J.F., van der Linden, D., Bakker, A.B., & Kompier, M.A. (2015). A multifaceted Investigation of the link between mental fatigue and task disengagement. *Psychophysiology* **52**, 305–315.

Langner, R. & Eickhoff, S.B. (2013). Sustaining attention to simple tasks: a meta-analytic review of the neural mechanisms of vigilant attention. *Psychol Bull* **139**, 870-900.

Mittner, M., Hawkins, G.E., Boekel, W., & Forstmann, B.U. (2016). A neural model of mind wandering. *Trends in Cognitive Sciences* **20,** 570–578.

Posner, M.I. & Rafal, R.D. (1987). Cognitive theories of attention and the rehabilitation of attentional deficits. in *Neuropsychological Rehabilitation*, (eds. Meier, M.J., Benton, A.L., & Diller, L.) 182-201 (Guilford Press New York USA).
